# Impact of the 2017 Child and Adult Care Food Program Meal Pattern Requirement Change on Menu Quality in Tribal Early Care Environments: The Food Resource Equity and Sustainability for Health Study

**DOI:** 10.1093/cdn/nzz094

**Published:** 2019-08-29

**Authors:** Susan B Sisson, Kaysha Sleet, Rachel Rickman, Charlotte Love, Alexandria Bledsoe, Mary Williams, Valarie Blue Bird Jernigan

**Affiliations:** 1 Department of Nutritional Sciences, University of Oklahoma Health Sciences Center, Oklahoma City, OK; 2 Center for Indigenous Health Research and Policy, Oklahoma State University, Tulsa, OK; 3 Department of Health Promotion Sciences, College of Public Health, University of Oklahoma Health Sciences Center, Tulsa, OK

**Keywords:** Native American, tribal, food program, preschool, young children, menu, dietary intake, community-based participatory research, CACFP, Early Care and Education

## Abstract

**Background:**

Native American (NA) children have a high prevalence of obesity contributing to lifespan health disparities. Dietary intake is important to promote healthy weight gain, growth, and development. In 2017, the USDA enforced changes to the Child and Adult Care Food Program (CACFP). The CACFP provides reimbursement to qualifying Early Care and Education (ECE) programs that serve foods that uphold the program's nutrition requirements.

**Objective:**

This study had the following 2 objectives: *1*) Describe a novel index to evaluate ECE menus based on revised CACFP requirements (accounting for food substitutions) and best practices for 3- to-5-y-old children, and *2*) analyze CACFP requirement and best practice compliance and nutrient changes in 9 NA ECE programs before and after enforcement of the revised CACFP requirements.

**Methods:**

This longitudinal study is within a larger community-based participatory research study. Menus and meals served were evaluated for 1 wk at each of 9 programs before and after enforcement of the revised meal patterns. Nutrient analysis, CACFP requirement and best practice compliance, and substitution quality were evaluated. Differences were determined using a paired *t*-test or Wilcoxon matched test. This trial was registered at clinicaltrials.gov as NCT03251950.

**Results:**

Total grams of fiber consumed increased (5.0 ± 1.2 compared with 5.9 ± 0.8 g, *P* = 0.04) and total grams of sugar consumed decreased (53.8 ± 12.6 compared with 48.4 ± 7.9 g, *P* = 0.024), although room for further improvement exists. Although total grams of fat remained unchanged, grams of saturated fat significantly increased (7.8 ± 1.4 compared with 10.5 ± 3.4, *P* = 0.041). Other nutrients remained unchanged. Overall CACFP requirement and best practice compliance scores improved, although this finding was not statistically significant. No significant changes in food quality associated with substitutions occurred.

**Conclusions:**

This study provides early evidence to support the beneficial impact of the revised CACFP requirements. Understanding barriers to compliance within rural NA communities would be an important next step in enhancing the health of vulnerable children.

## Introduction

In the United States, 9% of 2- to-5-y-old children were obese (1) and nearly 2% were extremely obese [≥120% of the 95th BMI percentile (BMI in kg/m^2^)] (2) in 2011–2014. Native American (NA) children have higher obesity rates than do other racial and ethnic populations (3–7), which may contribute to health disparities across the lifespan. Incidence of childhood obesity is highest during preschool years (8), and nearly 75% of children who are obese at 2 y old are predicted to be obese at age 35 (9). Obesity is associated with type 2 diabetes, certain cancers, and premature death (10). Risk factors for obesity in early childhood include low fruit and vegetable intake and low levels of moderate-to-vigorous physical activity; high intake of dietary fiber and limited intake of high-sugar foods are considered protective against childhood obesity (3, 11).

The majority of children (61%) in the United States attend Early Care and Education (ECE) programs (12). Children with employed mothers spend an average of 32 h/wk at these programs (13), and 50% to 66% of the calories consumed during the days that children are in ECE come from ECE-provided meals (14, 15). Children attending ECE programs are often served and consume foods high in fat (14, 16, 17), salt (17), sugar (17), and excessive protein (14, 18, 19). Furthermore, children in ECE do not eat enough grains or vegetables (11, 14, 16, 17, 20, 21), especially dark, nutrient-rich vegetables (16, 17, 20–22). Dietary intake is essential for optimal weight gain, growth, and development in early childhood. The USDA Child and Adult Care Food Program (CACFP) provides financial reimbursements to a variety of care facilities, including qualifying ECE programs that agree to abide by CACFP nutritional requirements. ECE programs participating in the CACFP prepare lunches that are more nutrient dense (23), include more fruits and vegetables and fewer sweets and sweetened beverages (24), include more whole grains (25), have more healthful feeding practices (25, 26), and more frequently comply with the Academy of Nutrition and Dietetics Benchmarks for Child Care (27) than do ECE programs that do not participate in the CACFP (23, 28–31). Furthermore, data from the Early Childhood Longitudinal Study–Birth Cohort, a national study conducted by the National Center for Education Statistics from 2001 to 2005, indicate that for low-income children, CACFP participation is associated with enhanced nutrient intake and lower odds of overweight and obesity (32).

**FIGURE 1:**
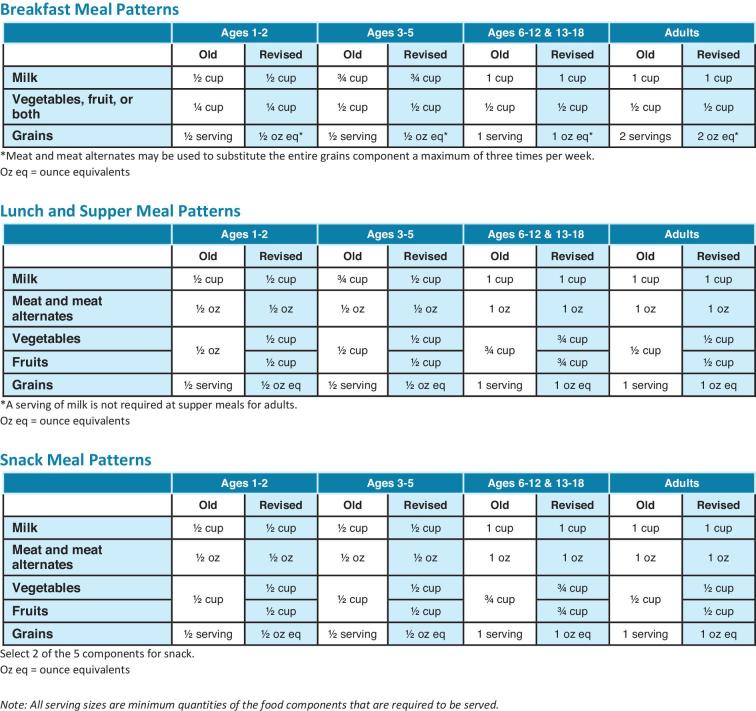
Old and revised (2017) CACFP meal pattern requirements. CACFP, Child and Adult Care Food Program.

In the fall of 2016, the USDA provided changes to the CACFP meal pattern requirements and best practices for the first time since 1968 (41). Enforcement of these changes began in the fall of 2017. Changes were made in an effort to align the CACFP meal pattern requirements more closely with the Dietary Guidelines for Americans (DGA) by limiting fruit juice and flavored milk, reducing fried and prefried foods, providing fruits and vegetables as snacks, serving dark green vegetables and legumes weekly, and providing family-style meal service (42). Old (pre-2017) and revised (2017) CACFP meal pattern requirements are shown in **Figure 1**. The availability of these modifications provides the opportunity to enhance the nutrition of children across the country. In 2017, there were 1.9 billion meals served, and ${\$}$3.5 billion dollars (${\$}$781/person) was paid to providers (43). Best practices include limiting juice and flavored milk, reducing fried and prefried foods, providing fruits and vegetables as snacks, serving dark vegetables and legumes weekly, and providing family-style meal service (41). However, there are variations in the fidelity with which programs implement the CACFP (33–36) and other nutrition policies (36, 37); these variations may compromise overall nutritional quality, and the current methods aimed at addressing this concern leave substantial room for improvement. A recently released Health Impact Assessment (HIA) predicts that the 2017 CACFP meal pattern requirements will improve children's dietary intake; however, there may be concomitant cost increases (44). Evidence from California lends optimistic support for the impact of policy change on meal quality in ECE, with improvements in water accessibility and decreases in whole milk and juice service with state policy changes in 2012 (45). There is limited understanding of how these changes will actually be implemented and affect menu and meal quality, especially in high-risk communities. These programs play important roles in early childhood nutrition. There is evidence that children attending ECE programs with healthier environments have healthier weight status (38). Thus, ECE programs are a viable environment for early obesity prevention interventions (39, 40).

Menu and meal quality have been operationally examined using a variety of metrics, including nutrient analyses (14, 17, 19, 20, 22, 23, 31, 33, 34, 46–54), number of target food groups (16, 17, 20, 22–24, 26, 34, 45, 51, 53–59), serving size compliance (22), tastes of food (57), food characteristics and origin (60), and calculated nutrient ratios (33). Few studies have examined how menus and meals served comply with the CACFP meal pattern requirements and best practices (22, 33–36), and different approaches were used in each study, although compliance was predominantly based on individual components rather than examination of the menu or meal as a whole. Lack of standardization limits researchers’ and practitioners’ ability to compare and aggregate findings easily, if at all. A standardized approach with a numerical score can be used more easily in statistical applications to evaluate the degree to which an ECE program complies with the revised 2017 CACFP meal pattern requirements and best practices, as well as allow comparisons across programs. A program's ability to make menu substitutions is important to adjust for availability, seasonality, and other issues within a program on a given day. Thus, the foods indicated on the menu may not reflect what is served during the meal. However, accounting for these substitutions adds a layer of complexity for nutrition practitioners and researchers, as these substitutions may alter the nutritional quality of meals and the degree to which programs adhere to CACFP requirements and best practices. Three previous studies have accounted for additions and omissions of foods served (35, 55, 56). However, the nutritional quality and comparability of these substitutions was not considered.

The purpose of this article is 2-fold: *1*) to describe a novel index constructed to evaluate ECE menus based on the revised CACFP meal pattern requirements (accounting for inevitable local-level food substitutions) and best practices for 3- to 5-y-old children and *2*) to analyze compliance with revised 2017 CACFP meal pattern requirements and best practices and nutrient changes of actual meals served (accounting for inevitable local-level food substitutions) to young children attending 9 ECE programs owned and operated by the Osage Nation before and after enforcement of the revised 2017 CACFP meal pattern requirements.

## Methods

### Study Design

This longitudinal study is part of a larger community-based participatory research study known as FRESH (Food Resource Equity and Sustainability for Health) that examines the impact of a gardening and education intervention on vegetable and fruit intake and food insecurity, BMI, and blood pressure among a cohort of NA families residing in the Osage Nation over a 6-mo period (registered at clinicaltrials.gov as NCT03251950). Nutrients of planned menus and actual meals (breakfast, lunch, and snacks) served to 3- to 5-y-old children at 9 ECEs were averaged across 1 wk both before and after enforcement of the revised CACFP meal pattern requirements and best practices in the fall of 2017. Evaluations were based on the ECE's compliance with the revised 2017 CACFP meal pattern requirements and best practices.

The FRESH study partnership began in 2013 and comprises a multisector group of representatives from the health (*n* = 2), education (*n* = 5), agriculture (*n* = 3), and government (*n* = 1) leadership within the Osage Nation, and public health and nutrition staff and faculty (*n* = 8) from the Oklahoma State University Center for Indigenous Health Research and Policy. The FRESH study was funded in 2016 and is led by the multisector tribal–university study executive committee. The measures discussed in this article represent those taken prior to the implementation of the FRESH intervention in the spring of 2018. Part of the FRESH intervention was training food preparers on the CACFP best practices in a 3-h, hands-on training session and providing tailored best-practice menus and recipes (61). Federal policy changes regarding the CACFP meal pattern requirements and best practices occurred during the planning and baseline data collection stages for the FRESH study and presented a prime opportunity to examine the impact of these modifications on a rural NA community's ECE programs.

The Osage Nation operates 9 ECE programs: 4 Head Start programs, 4 Wah-Zha-Zhi Early Learning Academies (WELAs), and 1 Osage Language Immersion School. While all but 1 of these ECE programs officially participates in the CACFP, they all share 1 central menu planning team, and a registered dietitian oversees the menus. These programs are located in the communities of Skiatook, Fairfax, Hominy, and Pawhuska, located on the Osage Nation reservation in Northeastern Oklahoma. Substitutions to the menu due to access limitations, such as available foods from vendors, spoilage, storage capacity, and food preparation staff needs are permitted (61). Enrollment at the Head Start programs, which have been in operation the longest of the Osage ECE programs, ranges from 19 to 95 children. The oldest program began operation in 1979, and the most recent program opened in 1985. All 4 WELA programs started more recently, from 2012 to 2017, and the Language Immersion program began in 2015. The WELA and Language Immersion programs served 12–34 children at the time of this study.

Menus and meals served to 3- to 5-y-old children were evaluated for each of the 9 programs at the following 5 time points throughout the study: *1*) September 2017 (before 2017 CACFP meal pattern requirements were enforced); *2*) October–November 2017 (after CACFP meal patterns changed, before food preparers’ training on best practices); *3*) February–March 2018 (after food preparers’ training on best practices, early in FRESH intervention); *4*) May 2018 (after food preparers’ training on best practices, late in FRESH intervention); and *5*) October 2018 (following the FRESH intervention). Only the first 2 time points representing meals immediately before (September 2017) and after (October/November 2017) enforcement of the revised 2017 CACFP meal pattern requirements are presented in this article. All programs operate with a 6-wk–cycle menu. Menus were collected from each program for the 6-wk cycle prior to and after enforcement of the revised 2017 CACFP meal pattern requirement changes, and foods served to children during week 5 were evaluated as described below. Research personnel traveled to each program at least twice, including at least 1 time during the evaluation week to assist with record keeping and recording and again after the evaluation week to collect data for all 5 days. Resources for this level of support for data collection could not be provided throughout the 6-wk cycle. At the time of the initial data collection, the programs were in the fifth week of their menu. For consistency, all subsequent data collection time points occurred on the fifth week of the menu cycle. Program food preparers provided recipes, food preparation styles, quantities, and brands of foods served during the week. Whenever possible, mixed dishes were broken into their individual ingredients and recorded. Research personnel took photographs of all of the brands and food labels to assist in the record keeping. Menus and actual foods served were evaluated using multiple approaches, including nutrient analyses, scores indicating compliance with the revised 2017 CACFP meal pattern requirements and best practices, and percentages and quality of menu substitutions. This study was approved by the University of Oklahoma Health Sciences Center Institutional Review Board (IRB), which acted as the IRB of record for the Osage Nation, per request of the Nation at the time of the study.

### Nutrient Analysis

All self-reported actual foods served at ECE programs during meals and snacks in week 5 of the 6-wk–cycle menu were entered into nutrient analysis software (ESHA Food Processor Nutrition Analyses Software) in conjunction with the USDA nutrient database. An average of all of the day's food that was served in 1 wk was used to make the study comparable to previous work (62, 63) and account for differences in the numbers of meals and snacks served across centers. Portion sizes were commonly not listed on menus and may not have been available for self-reported actual foods served. In such instances, the minimum required serving size from the revised 2017 CACFP meal pattern requirements for 3- to-5-y-old children was used, a methodology consistent that used in previous research (16, 33, 48, 62, 64, 65). A detailed list of the assumptions used to determine the nutrients are shown in Supplementary Material A [Description of Development and Child and Adult Care Food Program (CACFP) Compliance Tool]. Similarly, recipes, brands, and preparation methods are not available on a menu, and may not have been reported in adequate detail on the self-reported actual foods served, so a standardization process for food selection was used in concordance with previous research methods (48, 62). For instance, if chicken nuggets were reported without a brand, the same brand was used in each instance for that item it was not specifically reported. Should the brand of food not be available in the nutrient analyses software, the food label was searched online and a nutritionally similar brand was chosen. Nutrients of interest included calories, protein (grams), carbohydrate (grams), fiber (grams), soluble fiber (grams), sugar (grams), total fat (grams), saturated fat (grams), vitamin A retinol activity equivalents (micrograms), vitamin C (milligrams), vitamin D (micrograms), vitamin E alpha tocopherol (milligrams), folate (micrograms), calcium (milligrams), iron (milligrams), and sodium (milligrams). In addition to nutrients, total cups of MyPlate food group consumed were noted and included total grain (ounces), total vegetables (cups), total fruit (cups), total dairy (cups), and total protein (ounces, 1 oz = 28.3495 g).

### Compliance with Revised 2017 CACFP Meal Pattern Requirements and Best Practices

To assess compliance with the revised 2017 CACFP meal pattern requirements and best practices and change over time, a quantitative index was developed to evaluate the actual foods served. See Figure 1 for the old (pre-2017) and revised (2017) meal pattern requirements and best practices. Supplementary Material A describes in detail the index development process using the 2017 CACFP meal pattern requirements and best practices guidelines and included evaluations of components, variety, low-sugar cereal and yogurt, whole grains, and juice. Supplementary Material A also provides the index and shows an example of scoring. **Table 1** summarizes the scoring and categories. Some programs did not provide all meal and snack services and thus should not be penalized for not serving breakfast, for example, if a morning snack was provided. Thus, the points scored for a program were divided into the maximal points, for that program, to provide a percentage. Three percentages are presented: percentage of compliance to revised 2017 CACFP meal pattern requirements, percentage of compliance with revised 2017 CACFP best practices, and percentage of overall compliance (revised 2017 CACFP meal pattern requirements and best practices combined). Thus, each section of the compliance index, meal pattern requirements, best practices, and overall total, would have a possible score of zero to 100%.

**TABLE 1:** Summary of CACFP meal pattern requirement and menu and meal compliance scoring^1^

One-week menu (max 51 points)	Points
Requirements (max 26 points)	
Breakfast on the menu	1
Lunch on the menu	1
Snack on the menu	1
Breakfast	
Unflavored and low-fat milk on the menu	1
Vegetables on the menu	1
Fruit on the menu	1
Grain on the menu	1
Meat or meat alternate on the menu	1
No juice on the menu	1
Lunch	
Unflavored and low-fat milk on the menu	1
Vegetables on the menu	1
Meat or meat alternate on the menu	1
Fruit or second vegetable on the menu	1
Grain on the menu	1
No juice on the menu	1
Snack	
Sufficient components on the menu	1
Unflavored and low-fat milk on the menu	1
Meat or meat alternate on the menu	1
Vegetables on the menu	1
Fruit on the menu	1
Grain on the menu	1
No juice on the menu	1
General	
Low-sugar cereal on the menu	1
Low-sugar yogurt on the menu	1
≤1/d juice on the menu	1
≥1 whole grain/d on the menu	1
Best practices (max 25 points)	
Snack on the menu	1
Vegetables served for snack on the menu	1
Fruit served for snacks on the menu	1
Is 1 of 2 snack components a fruit or vegetable daily	1
Variety of fruit on the menu	1
Fruit on menu other than canned fruit, apples, banana, orange	1
Is fruit fresh or frozen on the menu	1
Majority of fruits fresh or frozen on the menu	1
Vegetables on menu other than peas, carrots, green beans, potatoes, tomato	1
Is fresh or frozen vegetables on the menu	1
Majority of vegetables fresh or frozen on the menu	1
≥1 serving dark green vegetables on the menu	1
≥1 serving red/orange vegetables on the menu	1
≥1 serving legumes on the menu	1
≥1 serving starchy vegetables on the menu	1
≤1/wk serving processed meat on the menu	1
Whole-grain foods served ≥ 2/d most days on the menu	1
Is cheese specified as low fat on the menu	1
Seasonal food items on the menu	1
Local produce on the menu	1
No sweet toppings on the menu	1
No yogurt candy mix-ins on the menu	1
No sugary beverages on the menu	1
No juice on the menu	1
≤1/wk serving prefried foods on the menu	1
Lunch observation meal (max 28 points)	
Requirements (max 8 points)	
No use of food as punishment or reward	1
Water availability	1
Unflavored and low-fat milk served	1
Vegetables served	1
Meat or meat alternate served	1
Fruit or 2nd vegetable served	1
Grain served	1
No juice served	1
Best practices (max 18 points)	
≥1 serving dark green vegetables served	1
≥1 serving red/orange vegetables served	1
≥1 serving legumes served	1
≥1 starchy vegetable served	1
Family style meal service	1
Whole-grain foods served	1
Low-fat cheese served	1
Seasonal foods served	1
Local produce served	1
Fruit served other than canned fruit, apples, banana, orange	1
Fruits served fresh or frozen	1
Vegetables served other than peas, carrots, green beans, potatoes, tomato	1
Vegetables served fresh or frozen and not prefried	1
No sweet toppings served	1
No yogurt candy mix-ins served	1
No sugary beverages served	1
No juice served	1
No prefried foods served	1
Food preparation (max 2 points)	
Lunch prep methods limited to steaming, baking, pressure cooking	1
Scratch (vs. processed food) preparation	1

^1^CACFP, Child and Adult Care Food Program; max, maximum.

### Substitutions

Programs are allowed to deviate from the menu and provide a substitution for a food component, should they choose to do so. While a substitution is not inherently adverse, substitutions with foods lower in nutrient-density would be undesirable. The percentage of food components substituted were calculated. Additionally, the substitution quality was classified as nutritionally equivalent; nutritionally superior, defined as higher nutrient-density; or nutritionally inferior, defined as poorer nutrient density. For example, chicken nuggets are a nutritionally equivalent substitution for fish sticks, whereas canned green beans are a nutritionally inferior substitution for fresh green beans due to the additional sodium present in canned vegetables. Fresh cantaloupe is a nutritionally superior substitution for canned fruit due to the likelihood of greater nutrients and no added sugars. Greater detail on these classifications can be found in Supplementary Material B (List and Details of Assumptions Used in Nutrient, Compliance, and Substitution Analyses for Menus and Foods Served).

Substitutions were calculated for *1*) each meal served during the week, *2*) each day, and *3*) total weekly substitutions. The number of food components that were served (e.g., 5 at lunch) was the denominator and the number of total substitutions (including additional components served) was the numerator, resulting in a percentage of total substitutions. For example, breakfast (3 components), lunch (5 components), and snack (2 components) have a total of 10 required components. If these 10 components were served for 5 days in the week, the denominator would be 50 total components. If there were 25 substitutions that week across all meals, then the numerator would be 25, yielding a percentage of total substitutions of 50% [(25/50) × 100].

### Data Analyses

Means, SDs, medians, and IQRs were calculated for all variables. Paired *t*-tests or Wilcoxon matched tests were used to determine nutrient differences in daily averages both before and after enforcement of the revised 2017 CACFP meal pattern requirements. Pearson's correlation coefficient was used to determine correlations between compliance scores and numbers of superior and inferior substitutions.

## Results

Meals served were reported for 2.5 ± 0.5 meals per program and for 4.9 ± 0.4 days per wk before (time point 1) and 2.6 ± 0.5 meals per program and 4.6 ± 0.5 days per wk after (time point 2) enforcement of the revised 2017 CACFP meal pattern requirements. No data collection occurred for the newest and smallest of the ECE programs during time point 1, since the program did not record any of the meals served during the target dates, leaving 8 ECE programs for pre- and postcomparison. Due to special events, scheduled in-service days, or school closures due to inclement weather during data collection time points, 1 program was open for 4 d, and the remaining 7 programs were open for 5 d during time point 1. During time point 2, 3 programs were open for 4 d, with the remaining 6 programs open for all 5 d.

Paired analyses before and after enforcement of the revised 2017 CACFP meal pattern requirements showed statistically significant improvements in mean ± SD total grams of fiber per (5.0 ± 1.2 compared with 5.9 ± 0.8 g, p = 0.04) and mean ± SD total grams of sugar per day (53.8 ± 12.6 compared with 48.4 ± 7.9 g, p = 0.024). Mean total grams of fat per day was unchanged, but mean ± SD total grams of saturated fat per day increased over time (7.8 ± 1.4 compared with 10.5 ± 3.4 g, p = 0.041). This last result warrants further investigation into what types of saturated fat increased (short, medium or long-chain), although that is not possible with current software sensitivity. None of the values of other measured nutrients changed significantly across the time points. Nutrient composition is shown in **Table 2**.

**TABLE 2:** Tribal ECE programs nutrient composition across all meals served in a week before and after enforcement of the 2017 CACFP meal pattern requirements^1^

		Time 1		Time 2			
Variable	Daily dietary reference intake values (66–68, 73)	Mean ± SD	Median ± IQR	Mean ± SD	Median ± IQR	*P* value	Cohen's *d*
Total kcals	1000–1600 based on age, sex, and physical activity	643.6 ± 140.8	655.6 ± 270.2	643.7 ± 106.6	655.7 ± 169.6	0.865^3^	0.00
Total protein, g	13 g for 1–3-y-olds	29.4 ± 7.2	28.2 ± 10.1	29.6 ± 4.4	31.3 ± 7.9	0.484^2^	0.03
	19 g for 4–8-y-olds						
Total carbohydrates, g	130 g for 1–3-y-olds and 4–8-y-olds	87.0 ± 21.8	89.0 ± 44.1	83.2 ± 12.4	85.4 ± 17.39	0.362^3^	0.22
Total fiber, g	19 g for 1–3-y-olds	5.0 ± 1.2	5.1 ± 1.9	5.9 ± 0.8	6.1 ± 1.6	0.040^3^	0.90
	25 g for 4–8-y-olds						
Total soluble fiber, g	Not specified	0.72 ± 0.3	0.73 ± 0.51	0.8 ± 0.2	0.8 ± 0.2	0.404^3^	0.32
Total sugar, g	Added sugars not to exceed 10–25% energy	53.8 ± 12.6	55.8 ± 24.1	48.4 ± 7.9	49.8 ± 11.8	0.024^3^	0.53
Total fat, g	30–40% energy for 1–3-y-olds	21.0 ± 3.9	20.7 ± 5.7	22.9 ± 5.5	20.8 ± 10.3	0.403^3^	0.40
	25–35% energy for 4–8-y-olds						
Saturated fat, g	No specified range other than to minimize consumption	7.8 ± 1.4	7.9 ± 2.6	10.5 ± 3.4	9.9 ± 5.9	0.041^3^	1.13
Vitamin A RAE, µg	300 µg for 1–3-y-olds	261.4 ± 80.3	291.9 ± 164.5	233.2 ± 50	252.7 ± 87.5	0.475^3^	0.43
	400 µg for 4–8-y-olds						
Vitamin C, mg	15 mg for 1–3-y-olds	34.9 ± 12.4	35.3 ± 20.2	30.6 ± 12.5	23.7 ± 24.8	0.523^3^	0.35
	25 mg for 4–8-y-olds						
Vitamin D, µg	15 µg for 1–3 and 4–8-y-olds	0.28 ± 0.2	0.29 ± 0.25	0.29 ± 0.2	0.2 ± 0.3	0.886^3^	0.05
Vitamin E alpha toco, mg	6 mg for 1–3-y-olds	1.4 ± 0.5	1.4 ± 0.56	1.1 ± 0.5	0.9 ± 0.9	0.523^3^	0.60
	7 mg for 4–8-y-olds						
Folate, µg	150 µg for 1–3-y-olds	77.9 ± 31.2	67.9 ± 35.4	70.7 ± 17.1	72.6 ± 29.1	0.603^3^	0.30
	200 µg for 4–8-y-olds						
Calcium, mg	700 mg for 1–3-y-olds	640.8 ± 139.8	593.9 ± 285.3	677.5 ± 123.9	724.8 ± 238.8	0.379^3^	0.28
	1000 mg for 4–8-y-olds						
Iron, mg	7 mg for 1–3-y-olds	3.6 ± 1.5	3.4 ± 2.8	3.5 ± 0.6	3.5 ± 1	0.803^3^	0.10
	10 mg for 4–8-y-olds						
Sodium, mg	800–1000 mg	1054.2 ± 299	1044.2 ± 605.7	1046.5 ± 255.8	957.6 ± 440.1	0.765^3^	0.03
Total grain, oz	3–5 oz based on age, sex, and physical activity	1.2 ± 0.2	1.2 ± 0.43	1.2 ± 0.3	1.2 ± 0.6	0.740^3^	0.00
Total vegetables, cups	1–1.5 cups for 2–8-y-olds based on age, sex, and physical activity	0.41 ± 0.16	0.41 ± 0.19	0.5 ± 0.2	0.5 ± 0.3	0.460^3^	0.50
Total fruit, cups	1–1.5 cups for 2–8-y-olds based on age, sex, and physical activity	0.85 ± 0.22	0.83 ± 0.41	0.8 ± 0.2	0.8 ± 0.3	0.201^3^	0.24
Total dairy, cups	2–2.5 cups based on age, sex, and physical activity	1.9 ± 0.4	1.8 ± 0.82	2 ± 0.4	2.2 ± 0.8	0.382^3^	0.25
Total protein, oz equivalents	2–5 oz equivalents based on age, sex, and physical activity	1.2 ± 0.56	1.2 ± 0.67	1.3 ± 0.3	1.2 ± 0.4	0.554^3^	0.23

^1^CACFP, Child and Adult Care Food Program; ECE, Early Care and Education; oz, ounces (1 oz = 28.3495 g); RAE, retinol activity equivalents.

^2^Wilcoxon test,

^3^Paired *t*-test.

**FIGURE 2:**
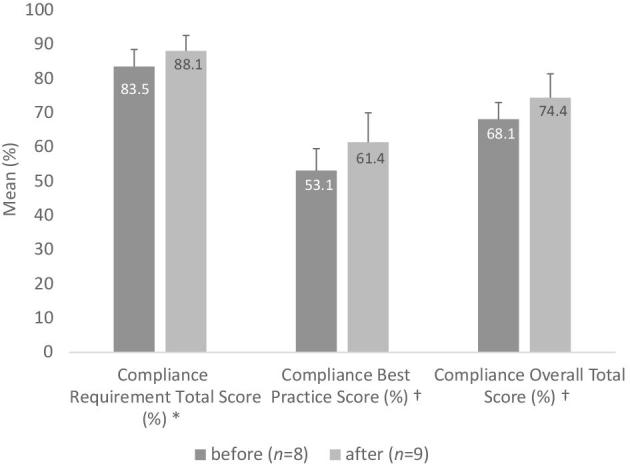
CACFP meal pattern requirements and best practices compliance (mean ± SD) in tribal Early Care and Education programs in Oklahoma before and after enforcement of the 2017 meal pattern revisions. CACFP, Child and Adult Care Food Program.

**FIGURE 3:**
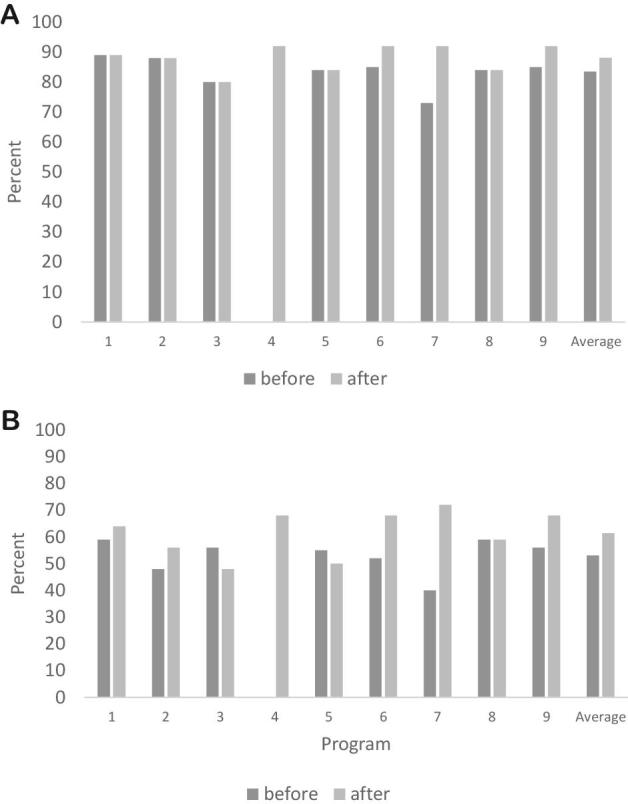
CACFP meal pattern requirement (A) and best practice (B) compliance by each tribal Early Care and Education Program in Oklahoma before and after enforcement of the 2017 meal pattern revisions. CACFP, Child and Adult Care Food Program.

Author-constructed indices about the revised 2017 CACFP meal pattern requirements and best practices did not produce statistically significant results, although a strong pattern of improvement emerged (**Figure 2**). While not statistically significant, **Figure 3**A shows that 4 out of the 8 (50%) ECEs had improved scores in the constructed index on CACFP meal pattern requirement compliance, and Figure 3B shows that 6 of the 8 (75%) ECEs has improved scores on the constructed CACFP best practice index. There were no significant changes in the percentage of superior, inferior, or equivalent local-level substitutions across the time points.

Indices for the revised 2017 CACFP meal pattern requirements, best practice recommendations, and overall total scores each increased (4.6%, 8.3%, and 6.3%, respectively). These Tribal ECE programs demonstrated approximately 83.5% compliance with revised 2017 CACFP requirements at time point 1 and 88.1% compliance at time point2. Compliance with CACFP best practices was understandably lower, at 53.1% at time point 1 and 61.4% at time point 2, since the old (pre-2017) meal pattern did not include guidelines for best practices.

Not all programs responded equally to the revised 2017 CACFP meal pattern requirement and best practice changes (Figure 3A and B). Regarding changes in compliance with CACFP meal pattern requirements, 5 programs remained about the same, 3 programs had small improvements, 1 program had substantial improvements, and 1 program moved from not having a menu at all to having a menu that met more than 90% of the revised 2017 CACFP meal pattern requirements. Regarding changes in compliance with CACFP best practice, 1 program remained the same, 4 had small improvements, 2 had major improvements, 2 had decreased best practice compliance, and the program without a menu moved to meeting nearly 70% of best practice guidelines.

**FIGURE 4:**
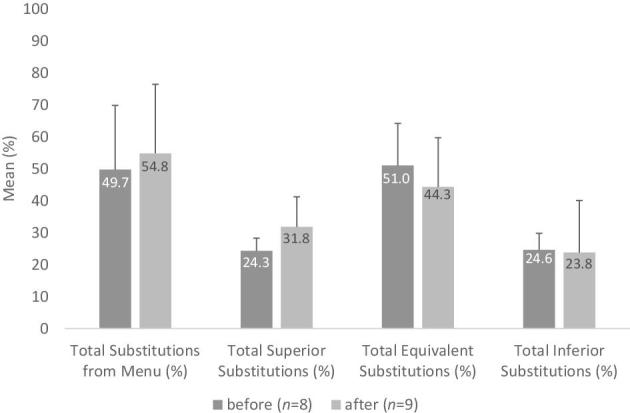
Tribal Early Care and Education programs menu substitution quality (mean ± SD) before and after enforcement of the 2017 CACFP meal pattern revision. CACFP, Child and Adult Care Food Program.

The mean ± SD percentages of substitutions across the observation weeks was 49.7% ± 20.1% and 54.8% ± 21.6% for time point 1 and time point 2, respectively. There were no statistically significant changes in the percentages of superior, inferior, or equivalent substitutions across the time points (**Figure 4**). Inferior substitutions decreased by 0.8%, equivalent substitutions decreased by 6.7%, and superior substitutions increased by 7.5%. In addition to substantial variance across programs, it appears that the percentage of superior substitutions slightly increased over time, while the percentages of equivalent substitutions slightly decreased. Inferior substitutions remained stable. There were no significant correlations between compliance scores and superior or inferior substitutions.

## Discussion

The main purpose of this study was to examine the impact of the revised 2017 CACFP meal pattern on the nutritional quality of menus and meals served to 3- to 5-y-old children in Tribal ECE programs in the FRESH study. This is the first study to examine the impact of the 2017 CACFP meal pattern requirements in ECE programs, and in tribal ECE programs in particular. A recent HIA on the impact of the updated CACFP meal pattern requirements projected increases in nutritional quality, variety of foods consumed, and consumption of underconsumed nutrients and reductions in consumption of sugar and saturated fat (44). In addition to changes in foods served, the HIA also predicted reductions in CACFP participation, and reduction in prevalence of overweight and obesity, as well as improvements in teacher and child attitudes toward healthy eating and decreases in program costs (44). While the timeline of the present study precludes evaluation of some of these hypothesized outcomes, our findings do support some findings that the introduction of 2017 meal pattern requirements led to decreased sugar and increased fiber served in ECE programs.

Our finding that compliance with CACFP requirements was higher than compliance with best practices is consistent with findings reported in the previous literature (22, 33–36), although methodological approaches varied greatly. To our knowledge, ours is the first study to assign a numerical value to compliance, rather than examine a proportion of components or policies. Furthermore, the demonstrated increases in compliance with the revised 2017 CACFP meal pattern requirements and best practice recommendations along with the increase in total overall score, indicate that this method is able to detect differences in menu quality related to policy change. Although none of the score changes were statistically significant, the changes were clearly in the expected direction, demonstrating positive potential for substantial nutritional improvements at scale. Improvement in the best practice score, in particular, indicated increased service of a variety of fresh and frozen vegetables and fruit, as opposed to canned varieties.

About half of the menu components were substitutions (49.7% before and 54.8% after the meal pattern change), which is slightly higher than previous studies reporting that meal and snack substitutions ranged between 24 and 48% (35, 55, 56, 59). Our finding that approximately 75% of the substitutions were nutritionally equivalent or better is an important addition to the growing body of literature. Previous studies did not include whether a substitution was nutritionally equal and instead simply determined whether foods were within the same food category (35, 55), the exact same food (59), or added/omitted (35, 55, 56). While the current analyses were limited by a small sample size, the increase in superior substitutions further indicates that the revised meal pattern can make an impact when food preparers are making quick decisions on menu modifications. Efforts to align foods served with those planned in the menu would enhance the ability of researchers and parents to better understand the nutritional quality of foods served to children. Food procurement can be challenging, especially in rural, tribal communities (61), and foods that are frequently substituted out can be replaced with those that are more accessible from vendors and local stores.

Significant increases in total fiber consumption and decreases in total sugar consumption were dietary improvements associated with implementation of the revised 2017 meal pattern requirements, supporting changes predicted in the HIA (44). These findings are encouraging, given the small sample size, and expected, as major changes to the CACFP meal pattern requirements included the elimination of grain-based desserts, required service of low-sugar cereal and yogurt, and required ECEs to serve at least 1 whole grain food per d (41). However, sugar intake was and remains high, and fiber was and remains low. Thus, room for improvement still exists. The DGA (66) and the WHO (67) recommend that added sugars not exceed 25% and 10%, respectively, of daily dietary intake. Daily caloric needs of preschool-age children range from 1000 to 1600 kcal/d based on age, sex, and physical activity level (68), and 50% to 66% of energy needs are met during childcare hours (14, 15). Although our data include added sugars as well as those naturally occurring in foods, sugar provided across meals at ECE dropped from 33% to 30% of energy provided with the new meal pattern. After enforcement of the revised 2017 CACFP meal pattern requirements, children were served 643 kcal including 48 g/d of sugar, which is 194 kcal, equating to 30% of energy provided in the form of sugar, which exceeds recommendations. Similarly, the DGA (66) recommend 19 g of fiber for 1- to 3-y-old and 25 g of fiber for 4- to 8-y-old children. Fiber provided to these children across 2.5–2.6 meals increased from 20–26% to 24–31% of daily fiber needs to 5.9 g. Given the volume of time spent in ECE, 50–66% of fiber needs should be met, resulting in a minimum of 10–13 g, whereas children were being served half this recommendation.

An unanticipated change was an increase in saturated fat by approximately 2.7 g per lunch, which was contrary to expectations described in the HIA (44). While some short- and medium-chain saturated fats are associated with positive health outcomes (69), the DGA recommend that intake of saturated fatty acids remain low. Additionally, full-fat dairy foods may be associated with health benefits, specifically in NA populations (70); however, DGA and the CACFP support service of low-fat dairy foods. Further, examination of the types of fatty acids in these meals was outside the scope of this study. Thus, we cannot provide a clear explanation for this observation. Given the increase in fiber following enforcement of the revised 2017 CACFP meal pattern requirements, it was surprising to observe almost no change in total ounces of grains or cups of produce.

Dietary recommendations for vegetables and fruit for 2- to 8-y-old children is 1 to 1.5 cups each (68). At ECE programs, children were served approximately 0.5 cups vegetables and 0.9 cups fruit throughout the day. With ECE programs serving more varieties and higher volumes of produce than families (71, 72), efforts can be made to further increase volume and variety at ECE programs to ensure optimal intake for young children. The amount of protein served to children was higher than the DGAs recommendations (30 g/d compared with 13–19 g/d) (66), although within the acceptable macronutrient distribution ranges of 10–30% of energy at 18% of energy. Relative and absolute volumes of protein were similar to those in previously reported of meals served in ECE (11, 19, 51). Percentage of energy from fat was also within acceptable macronutrient distribution ranges of 25–35% (66) and similar to the values reported for other studies (11, 19, 51). Sodium served to children across the 2.5 meals included in analyses exceeded the daily allowance of 800–1000 mg (73). Given a reliance on processed foods, this finding is not wholly unexpected, although it is concerning, as these children will likely eat other, less healthy meals outside the ECE environment (71, 72). The amount of vitamin D, vitamin E, folate, and iron provided was below recommendations (66). However, 2.5 meals at ECE programs appear to be sufficient in providing the majority of daily needs for vitamin A, vitamin C, and calcium (66). Many people, and NA in particular, are deficient in vitamin D (74), supporting the need for vitamin D–fortified milk and a variety of vegetables and foods fortified with vitamin E.

The range in variability in CACFP compliance and menu substitutions appears to increase from time point 1 to time point 2. When examining changes in CACFP meal pattern requirements and best practice compliance at each ECE, some programs had greater improvement than others. As these were federal requirements, we hypothesize that this variability in implementation, even with centralized menu planning, was limited by external barriers and internal capacity. External barriers restrict food access, such as limited availability and quality of local grocers in all 4 communities, and limited numbers of vendors who will deliver to the rural communities. Internal capacity restrictions noted by the cooks at the schools included physical space for fresh produce storage and preparation and limitations in staffing to prepare foods from scratch.

The strengths, limitations, and future opportunities of this analysis warrant discussion. To our knowledge, this is the first article to describe the impact of the revised 2017 CACFP meal pattern requirements and in rural, tribally affiliated ECE programs. The development of the CACFP compliance scores and substitution quality evaluation indices is innovative; the process allowed deeper understanding and comparison of ECE menus to federal standards and best practice recommendations. These indices can be utilized by other investigators in future studies as a standardized approach to quantify menu compliance and substitution quality. It is important to note that the index determines compliance with the CACFP meal patterns requirements and best practices and does not in and of itself evaluate nutritional quality or aspects of cutting edge research regarding nutrients and components. While the sample size is small, only 9 programs, the findings of this case series study can be used to inform and guide future larger-scale evaluations. Regardless of the limited sample size, statistically significant findings were observed for some variables, with others not reaching statistical significance, but demonstrating movement in the anticipated direction. Continued efforts to provide optimal meals include reducing sodium, sugar, and protein, while increasing fiber and vitamins from fruit and vegetables. Substantial variability in degree of capacity at individual programs, which may be outside the program's control, may limit a program's ability to fully adhere to revised 2017 CACFP requirements and best practices. Such variability and context should be included in future research.

## Conclusion

To our knowledge, this is the first study to examine the impact of enforcement of the revised 2017 CACFP meal pattern requirements on nutritional quality of menus, compliance with CACFP meal pattern requirements and best practices, and quality of menu substitutions in rural, tribally owned and operated ECE programs. This study provides early evidence to support the beneficial impact of the revised 2017 CACFP meal pattern requirements and best practices on nutritional quality, compliance with requirements and best practices, and menu and meal substitutions that are of equivalent or higher nutritional quality. Although few outcomes were statistically significant, the majority were in the hypothesized direction. Continued improvement regarding nutrient density and lower sodium in meals provided is necessary to strive toward meeting optimal nutrition for 3- to 5-y-old children attending ECE programs across the United States.

## Notes

Funded by the National Institute on Minority Health and Health Disparities (R01MD011266). The funding agency did not participate in the study design, data collection, analysis, decision to publish, or preparation of the manuscript.

Author disclosures: SBS, KS, RR, CL, AB, MW, and VBBJ, no conflicts to disclose.

This article appears as part of the supplement “Proceedings of the Third Annual Conference on Native American Nutrition,” sponsored by the Shakopee Mdewakanton Sioux Community's Seeds of Native Health campaign through a gift to the University of Minnesota. The guest editors of the supplement, Treena Delormier and Mindy S Kurzer, have no conflicts of interest. The opinions expressed in this publication are those of the authors and are not attributable to the sponsors or the publisher, Editor, or Editorial Board of *Current Developments in Nutrition*. Publication costs for this supplement were defrayed in part by the payment of page charges.

Abbreviations used: CACFP, Child and Adult Care Food Program; DGA, Dietary Guidelines for Americans; ECE, Early Care and Education; FRESH, Food Resource Equity and Sustainability for Health; HIA, Health Impact Assessment; IRB, institutional review board; NA, Native American; WELA, Wah-Zha-Zhi Early Learning Academy.
